# An Ultra-High-Energy Density Supercapacitor; Fabrication Based on Thiol-functionalized Graphene Oxide Scrolls

**DOI:** 10.3390/nano9020148

**Published:** 2019-01-24

**Authors:** Janardhanan. R. Rani, Ranjith Thangavel, Se-I Oh, Yun Sung Lee, Jae-Hyung Jang

**Affiliations:** 1 School of Electrical Engineering and Computer Science, Gwangju Institute of Science and Technology, Gwangju 61005, Korea; ranijnair@gmail.com (J.R.R.); ohseia@naver.com (S.-I.O.); 2 Faculty of Applied Chemical Engineering, Chonnam National University, Gwangju 61186, Korea; ranjith.cecri@gmail.com (R.T.); leeys@jnu.ac.kr (Y.S.L.); 3 Research Institute for Solar and Sustainable Energies, Gwangju Institute of Science and Technology, Gwangju 61005, Korea

**Keywords:** EDLC, rGO scrolls, thiol functionalization, supercapacitor, energy and power density

## Abstract

Present state-of-the-art graphene-based electrodes for supercapacitors remain far from commercial requirements in terms of high energy density. The realization of high energy supercapacitor electrodes remains challenging, because graphene-based electrode materials are synthesized by the chemical modification of graphene. The modified graphene electrodes have lower electrical conductivity than ideal graphene, and limited electrochemically active surface areas due to restacking, which hinders the access of electrolyte ions, resulting in a low energy density. In order to solve the issue of restacking and low electrical conductivity, we introduce thiol-functionalized, nitrogen-doped, reduced graphene oxide scrolls as the electrode materials for an electric double-layer supercapacitor. The fabricated supercapacitor exhibits a very high energy/power density of 206 Wh/kg (59.74 Wh/L)/496 W/kg at a current density of 0.25 A/g, and a high power/energy density of 32 kW/kg (9.8 kW/L)/9.58 Wh/kg at a current density of 50 A/g; it also operates in a voltage range of 0~4 V with excellent cyclic stability of more than 20,000 cycles. By suitably combining the scroll-based electrode and electrolyte material, this study presents a strategy for electrode design for next-generation energy storage devices with high energy density without compromising the power density.

## 1. Introduction

The growing demand for energy storage systems in electric vehicles, load-leveling, and portable electronic devices has stimulated research into high-density electrochemical energy storage technologies that can deliver high power for long periods of time [1,2,3]. Electrochemical double-layer supercapacitors (EDLCs) have attracted considerable interest for such applications, due to their high specific power density and long cycle life [4,5]. The major drawback of EDLCs is their low energy density, which lies in the range of 3~5 Wh/kg [6,7], two orders of magnitude lower than that of the commercial lithium-ion batteries. Supercapacitors that can deliver high-energy density without sacrificing power density are critically needed for practical applications, such as hybrid electric vehicles [8], industrial forks [4], wind turbine energy storage [9], mobile electronics [1], fuel cells [10], regenerative braking [11], and power supply devices [12], etc. Several materials, including polymers [13,14,15] or reduced graphene oxide (rGO)-based materials have been introduced to overcome the EDLCs’ low energy density issue. Among them, rGO-based EDLCs have been reported with high power density, high charge/discharge rates, and long cycle life performance. However, even though tremendous progress has been achieved in developing rGO-based EDLCs with high energy density using hydrazine reduction (85.6 Wh/kg) [16], microwave exfoliation (70 Wh/kg) [17], and other processes, their energy density values are still significantly lower than the level needed for many practical applications.

Two of the major drawbacks of the rGO-based EDLCs that lead to the lower energy density values are the restacking of rGO layers and the low electrical conductivity of rGO. The agglomeration and restacking effects lead to a reduced electrochemically active surface area and interlayer spacing, with the result that ions in the electrolyte become less accessible, which in turn leads to the low energy density of the rGO-based EDLCs. In order to effectively implement EDLCs, their specific energy storage capabilities must be further improved. Energy density can be significantly enhanced by (1) preventing the restacking of rGO sheets, (2) increasing the electrical conductivity of the rGO sheets, and (3) creating a narrow pore size distribution in the synthesized rGO electrode.

In the present study, in order to prevent restacking and to increase conductivity, we synthesized nitrogen-incorporated, thiol-functionalized rGO scrolls (hereafter referred to as NTGS). The NTGS have unique three-dimensional, interconnected networks with a continuous porous structure, narrow pore size distribution, and a large surface area, making them potentially excellent electrode materials for high-performance EDLCs. The NTGS represent a promising new design strategy for optimizing the electrochemical performance of EDLC materials. Suppressing the sheet restacking yields a highly open and porous structure that creates continuous ion transport channels and allows electrolyte solutions to easily access the surfaces of individual graphene sheets.

Thiol-functionalized groups are composed of sulfur and hydrogen atoms (–SH). By functionalizing rGOs with a thiol (–SH) group, it is possible to tailor the physical and chemical properties of the rGOs. The synthesized NTGS show high electrical conductivity, which is attributed to (i) the orbital overlap between sulfur 3s and 3p, with π- orbitals in the rGO sheets, and (ii) the presence of lone pair electrons in nitrogen. The chemical bonding between sulfur and carbon is stronger than the van der Waals force between adjacent π−π stacked rGO layers. Moreover, additional states are formed in the conduction band of the rGO because of the thiolation process. Furthermore, the nitrogen atoms provide additional free electrons to the conduction band. The two physical and chemical properties of the NTGS result in significant enhancement of the electrical conductivity of the electrode.

In the present study, we achieve high energy density by synthesizing the unique scroll structure, which provides a short and rapid transport pathway for electrons, leading to the outstanding performance of the cell. The fabricated NTGS-based EDLC bridges the energy density gap between conventional batteries and supercapacitors. The fabricated supercapacitor exhibits a very high energy/power density of 206 Wh/kg (59.74 Wh/L)/496 W/kg, at a current density of 0.25 A/g, and a high power/energy density of 32 kW/kg (9.8 kW/L)/9.58 Wh/kg at a current density of 50 A/g, and excellent stability (>20,000 cycles).

## 2. Experiment Section

### 2.1. Preparation of Reduced Graphene Oxide and Thiol-Functionalized Reduced Graphene Oxide Scrolls

We synthesized GO using the modified Hummer’s method [18] by oxidizing graphite powder. Two-week-long dialysis was performed to completely remove the metal ions. The resulting GO solution was finally dried at 90 °C overnight to obtain GO powder. Then the powder was mixed well in an agate mortar for 30 min, followed by an annealing at 800 °C in ambient nitrogen for one hour. This rGO powder was used to fabricate the rGO cell.

For the preparation of thiol-functionalized rGO scrolls, the thiol-functionalized rGO powder (UniNanoTech Co., giheung-gu, Korea) was dispersed in 30 mL of water, and this mixture was suspended in water by sonication for 8 h, using an ultrasonic reactor operating at a frequency of 33 kHz. The mixture was centrifuged at 12,000 rpm for 15 min, which resulted in a homogenous suspension. Repeated sonication and centrifugation were carried out multiple times. The resulting solution was finally dried at 90 °C overnight to obtain the powder. The powder was mixed well in an agate mortar for 30 min, followed by an annealing at 800 °C in ambient nitrogen for one hour. The resulting powder consisted of nitrogen-incorporated, thiol–functionalized rGO nanoscrolls (NTGS).

The morphological, structural, and compositional properties of the NTGS powder were investigated using high-resolution transmission electron microscopy (HRTEM)/selective area electron diffraction (SAED) (JEM.ARM.200F), Raman spectroscopy (inVia Raman microscope), ultraviolet photoelectron spectroscopy (UPS), and X-ray photoelectron spectroscopy (XPS) measurements. The measurement of the nitrogen adsorption isothermal was carried out at 77 K by a low-temperature, nitrogen adsorption surface area analyzer (ASAP 2020, Micromeritics Ins., Norcross, GA, USA).

### 2.2. Cell Fabrication

Composite anodes were formulated with 80% active materials (NTGS or rGO), 10% Ketzen black (KB), and 10% teflonized acetylene black (TAB) using ethanol. The slurry was then pressed on a nickel mesh current collector with a 200-mm^2^-area, and dried at 160 °C for 5 h in a vacuum oven.

To evaluate the electrochemical performance of the NTGS electrodes, supercapacitors were assembled with symmetrical cell geometry. The test cells were constructed in an argon-filled glove box by compressing the NTGS and rGO electrodes, and the devices were fabricated with an ionic liquid EMIMBF_4_ electrolyte. The mass loading of electrodes is 2.5 mg cm^−2^, and the thickness of the working electrode is 57 μm. In the present study, stainless steel is used as the current collector, because in a large operating voltage window, the current collector needs to be more stable and non-corrosive in nature to maintain a stable cycling performance.

The electrochemical performance of the supercapacitor cells in the form of CR2032 coin cells was measured in the range of 0~4 V using a battery tester (WBCS 3000, Won-A-Tech, Seoul, Korea). The cyclic voltammetry (CV) was carried out at scan rates ranging from 10 mV/s to 200 mV/s, and the Galvanostatic charge/discharge (GCD) cycling of the cells was performed at current densities ranging from 0.26 A/g to 50 A/g.

## 3. Results and Discussion

**Figure 1:**
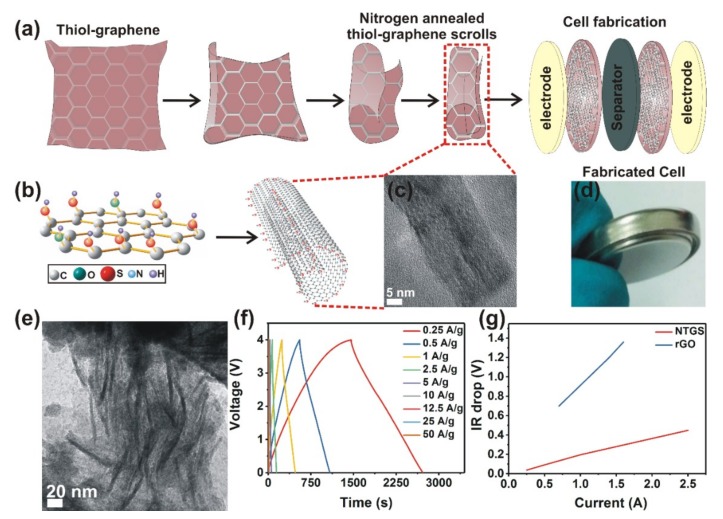
(**a**) Schematic showing the formation of the thiolated reduced graphene oxide (rGO) scrolls and cell fabrication, (**b**) structure of the planar N-doped thiol rGO and N-doped thiol rGO scroll, (**c**) high-resolution transmission electron microscopy (HRTEM) image of a single scroll, (**d**) digital image of a fabricated cell, (**e**) TEM image of an interconnected NTGS powder sample, (**f**) galvanostatic charge–discharge curves of the NTGS cell, and (**g**) ohmic voltage (IR) drop for the nitrogen-incorporated, thiol-functionalized rGO scrolls (NTGS) and rGO cells. The IR drop curve shows electrical conductivity was significantly enhanced in the NTGS compared to pure rGO.

Figure 1a illustrates the NTGS formation procedure. The NTGS samples were formed by rolling thiolated rGO layers in one or more directions. Scrolling occurs because of the graphene oxide’s (GO’s) planar structure, which is extremely unfavorable to maintain. A detailed discussion regarding the formation of the GO scrolls has been reported in our previous work [19].

The driving force for the scrolling of the thiolated GO is the energy difference between the total surface energy of the system and the elastic energy associated with thiolated GO bending [20,21]. When the temperature increases during annealing, the GO layer bends to minimize the total surface energy of the system by reducing the exposed surfaces of the thiolated GO layers, and the subsequent rolling of the graphene oxide is driven by the reduction of the total area of the exposed GO surface. Consequently, the total surface energy of the system is minimized by tightly wrapping adjacent graphene layers of the scroll together, where the layers are interconnected to each other. 

The morphology of the NTGS powder samples was analyzed using HRTEM images, as shown in Figure 1c,e. The HRTEM images of the NTGS clearly reveal the interconnected scroll structure. Additional TEM/HRTEM and selective area electron diffraction (SAED) images are shown in the Supplementary Figure S1. The TEM images show that the lateral diameter of the scrolls is in the range of 20~60 nm. The TEM images in Figure 1e indicate a high-quality interconnected carbon nanostructure, with randomly oriented rGO scrolls of various diameters and lengths that are connected and assembled together. Furthermore, the electron diffraction pattern in the high-magnification TEM image shows that the NTGS retain the highly crystalline graphitic structure (Figure S1i).

The Raman spectrum was also recorded and analyzed to identify the formation of scroll structures. The Raman spectra of the NTGS shown in Supplementary Figure S2a clearly show that the scrolls are significantly different from those of the planar graphene. The inset of Figure S2a shows the Raman spectra in the lower vibrational frequency range. The Raman spectrum of the scrolled GO differs significantly from that of flat GO. The high-degree curvature in the scrolled GO causes the appearance of low-frequency radial breathing-like (RBLM) modes. The most important feature in the Raman spectrum of Carbon nanotubes (CNTs) is the radial breathing mode (RBM), which is usually located between 75 and 300 cm^−1^. Figure S2a shows RBM modes around 100 and 172 cm^−1^, which again confirms the tubular structure of the GO scrolls in our samples. Also, the bands indicating the formation of scrolls are at 920, 1800, and 2000 cm^−1^, which are not observed in planar graphene/rGO [22,23]. The band observed at 630 cm^−1^ can be assigned to the C−S bond, confirming that there is strong interaction between carbon and sulfur [24]. The band observed at ~1280 cm^−1^ is consistent with sidewall functionalization, due to thiol-functional groups [25]. More explanations and the band assignment are given in the Supplementary Information. The functionalization of the thiol groups was also confirmed by X-ray photoelectron spectroscopy (XPS) analysis. The C1s, S2p, and N1s XPS spectra were fitted and deconvoluted, as shown in Supplementary Figure S2b–d. The XPS spectra clearly confirmed the strong chemical interaction between C–N and C–S in NTGS. The peak assignments and explanations are given in the Supplementary Information.

The electrochemical Galvanostatic charge/discharge (GCD) performance of the NTGS cell (in the voltage range between 0 and 4 V) was recorded at current densities ranging from 0.25 to 50 A/g (Figure 1f). The GCD curves clearly indicate excellent performance (The fabricated supercapacitor exhibits a very high energy /power density of 206 Wh/kg/496 W/kg, at a current density of 0.25 A/g and a high power/energy density of 32 kW/kg/9.58 Wh/kg at a current density of 50 A/g, specific capacitance of 360 F/g (104.4 F/cm^3^) as well as the double layer capacitive behavior of the fabricated NTGS cell). It is worth pointing that the cell can withstand a current density of up to 50 A/g.

The devices were fabricated with ionic liquid EMIMBF_4_ electrolyte. Compared with aqueous and organic electrolytes, ionic liquids (IL) are ideal electrolytes for supercapacitors because of their unique properties, such as high thermal stability and low flammability, which can improve the working temperature range and safety of supercapacitors. In particular, the wide electrochemical windows greater than 4 V can contribute greatly to improving the energy density of supercapacitors. A major drawback of the aqueous and organic electrolytes are safety issues, such as volatility and flammability. In addition, the working voltage window is very low, such as 1.2 V and 2.7 V for the aqueous and organic electrolytes, respectively. On the other hand, ILs are attractive electrolytes that can be an alternative to the conventional organic electrolytes because of their negligible vapor pressures, low flammability, and high electrochemical stability [26,27,28].

The GCD performance of the rGO cell is shown in the Supplementary Information (Figure S3). The rGO cell delivered a maximum energy density of 70 Wh/kg. The gravimetric specific capacitance (*C_gra_*), the gravimetric energy, and power density were calculated using the following Equations [29,30]:

(1) $$C_{gra}=4\frac{i\Delta t}{m\Delta V}$$

(2) $$E_{gra}=\frac{1}{8}C_{gra}\Delta V^{2}$$

(3) $$P_{gra}=\frac{E_{gra}}{t}$$

where *i, m,* Δ*t*, and Δ*V* are the applied current (A); mass (g) of the active material, including both the anode and cathode in the cell; discharge time; and potential window, respectively.

The volumetric specific capacitance (*C_vol_*) _can_ be calculated using the following equation:

(4) $$C_{vol}=C_{gra}\times \rho$$

The volumetric power density (P*_vol_*) and volumetric energy density (E*_vol_*) of the supercapacitor can be calculated as follows.

(5) $$E_{vol}=E_{gra}\times \rho$$

(6) $$P_{vol}=P_{gra\;}\times \rho$$

The packing density of total electrode material (*ρ*) was calculated according to the previous report, and the value is 0.29 g/cm^3^ [31].

The NTGS cell delivered a maximum capacitance of 360 F/g (104.4 F/cm^3^), an energy density of 206 Wh/kg (59.74 Wh/L), and a corresponding power density of 496 W/kg at a current density of 0.25 A/g. The cell delivered an energy density of 9.58 Wh/kg at a maximum power density of 32 kW/kg (9.8 kW/L) and at a current density of 50 A/g. Considering that the rGO cell delivered a maximum energy density of only 70 Wh/kg, the NTGS cell delivered three times higher energy density compared with the rGO cell.

A significant increase in the energy density of the NTGS electrode was observed, compared with those of other very recently reported graphene- and polymer-based supercapacitors. The gravimetric energy density of the NTGS electrode was higher than those of other very recently reported values, such as the conjugated indole-based macromolecule (30 Wh/kg) [32], reduced graphene oxide/mixed-valence manganese oxide composite (50 Wh/kg) [33], hollow particle-based nitrogen-doped carbon nanofiber (11 Wh/kg) [34], porous Ni_3_S_2_/CoNi_2_S_4_ three-dimensional (3D)-network structure (62.2 Wh/kg) [35], interlaced Ni(OH)_2_ nanoflakes wrapped in zinc cobalt sulfide nanotube arrays (75.5 Wh/kg) [36], honeycomb-carbon frameworks (55 Wh/kg) [37].

The enhancement in energy density of the NTGS cell over that of the GO cell can be attributed to (i) the higher conductivity of the NTGS electrode, and (ii) the scroll structure of NTGS, which eliminates restacking of the rGO and increases the electrolyte/electrode contact surface area. The cell resistance of the NTGS and rGO cells was confirmed by the IR drop against the discharge current, as shown in Figure 1g. The IR drop is calculated from the GCD curve, as shown in the previously published paper [38]. The IR drop in the GCD curve at a current density of 0.25 A/g is shown in Supplementary Information (Figure S4). It is worth noting that our NTGS cell exhibited resistance values that were only 1/6 that of the pure rGO cell. In supercapacitors, charge storage occurs via ion transportation and the intercalation of ions into the active material. Therefore, higher electrical conductivity or lower resistance of the active material results in higher capacitance values. The NTGS cell exhibited six times higher conductivity than the pure rGO cell, which in turn resulted in the higher capacitance and the energy density values. Moreover, the higher contact area and narrow pore size distribution provided by the peculiar scroll structure resulted in an enhancement of the electron transfer process between the electrode and electrolyte.

The enhanced electrical conductivity, higher specific area, and the narrow pore size distribution of NTGS samples were further investigated using ultraviolet photoelectron spectroscopy (UPS) and Brunauer–Emmett–Teller (BET) surface area measurements.

**Figure 2:**
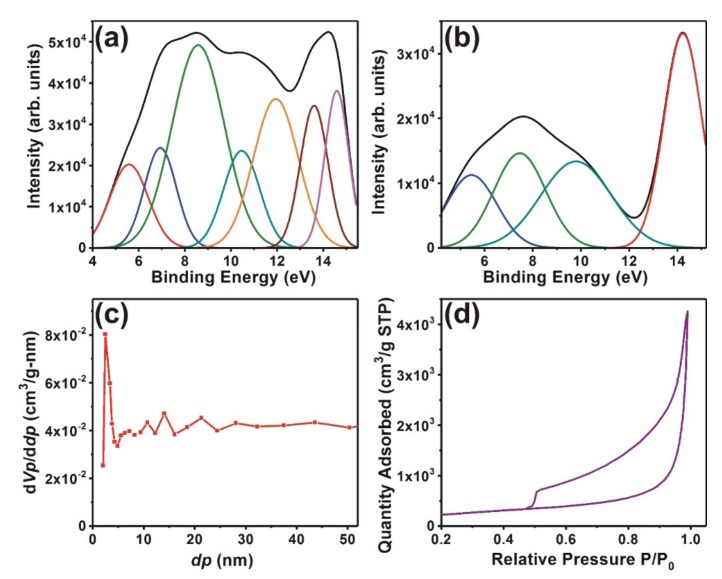
Fitted He-I ultraviolet photoelectron spectroscopy (UPS) spectra of (**a**) NTGS and (**b**) rGO powders after background subtraction. (**c**) Pore-size distribution and (**d**) nitrogen adsorption/desorption plot at 77.4 K of the NTGS sample.

Detailed study of the electronic structures of the powder samples was carried out using valence band UPS spectroscopy. UPS was performed in an ultrahigh vacuum (UHV) chamber with an He–I resonance line (*hν* = 21.22 eV) as an excitation source. As UPS sources can excite only valence band electrons, UPS spectroscopy can provide information about the valence band electrons responsible for bonding. The Fermi level position is referred at binding energy of 0 eV. UPS spectra for the NTGS and rGO samples were measured in the binding energy, ranging from 0 to 20 eV, as shown in Figure S5. This UPS spectrum reveals valence band energy levels of NTGS and rGO powders with respect to the source emission line (He1α; 21.22 eV). Figure 2a corresponds to the fitted He–I UPS spectra of the samples after subtracting background noise in the binding energy ranging from 4 to 15 eV. This is because the peaks corresponding to the graphene oxide appear in this range.

The fitted peaks and their corresponding assignments are shown in Supplementary Table S1. For comparison, the UPS spectra of pristine rGO samples were also recorded and deconvoluted, as shown in Figure 2b.

The UPS spectra of the rGO exhibited peaks at 5.4, 7.3, 9.9, and 14.3 eV. The UPS spectra of NTGS also showed similar peaks at 5.4, 7.0, and 14.6 eV. A noticeable difference was the additional 8.6, 10.5. 11.8, and 13.7 eV peaks in the NTGS UPS spectra. Interestingly, the peak observed at 9.9 eV in rGO, which was attributed to the orbital interaction between C2s and C2p, shifted towards a lower binding energy—8.6 eV in the NTGS [39]. We believe that this shift occurred because of the presence of lone pair electrons supplied by nitrogen. The additional peaks observed at 10.5, 11.8, and 13.7 eV correspond to C2p–S2p, C2p–S3s, and C2p–N2p interactions, respectively [39,40]. The UPS analyses confirmed that there were mixtures of the main orbital types of carbon and sulfur. The thiol functional group significantly improved the electrical conductivity because of the orbital overlap between sulfur 3s and 3p, with π- orbitals of carbon in the graphene sheets. The hybridization between carbon and sulfur increased the local charge density and the hybridization between S3p, S3s, and C2p states, resulting in the formation of impurity energy levels near the Fermi level, leading to higher conductivity. The hybridization between S3p states and C3s states also contributed to the formation of impurity energy levels.

The BET surface area measurement results are shown in Figure 2c,d. The interconnected NTGS exhibited a narrow pore size distribution, with an average pore diameter of 2.5 nm, as shown in Figure 2c. The scrolls had uniformly distributed mesopores with a narrow pore size distribution, resulting in easy accessibility for the electrolyte ions. Nitrogen adsorption/desorption plots of the samples at 77.4 K showed a type IV adsorption isothermal curve with a hysteresis loop (Figure 2d).

The interconnected NTGS had a large specific surface area of 803 m^2^/g. The higher specific surface area provided by the nanoscrolls resulted in an increase in the number of sites actually accessible to electrolyte ions, which enhanced the electron transfer process between the electrode and electrolyte. This enhancement of the electron transfer process gives rise to higher energy density. The high-surface-area NTGS with well-defined pores provides fast electronic and ionic conducting channels, making the material ideal for electrodes in supercapacitor applications.

**Figure 3:**
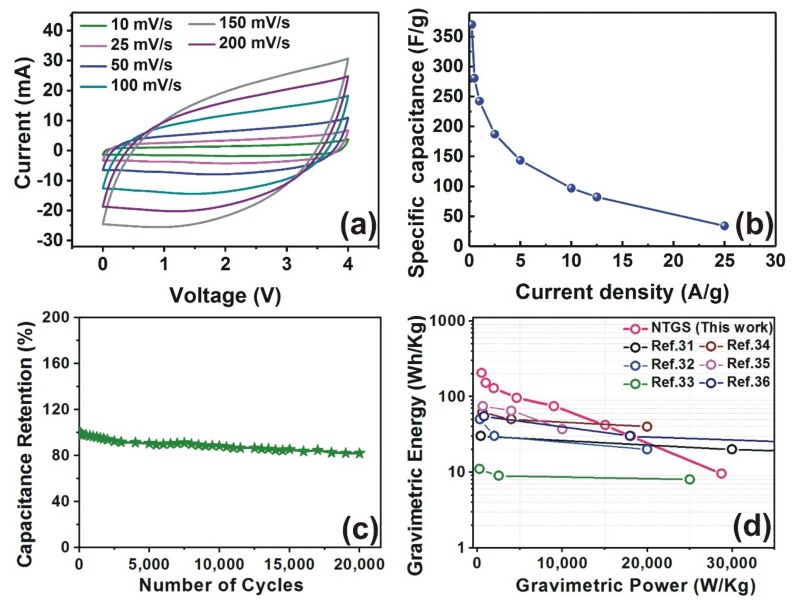
(**a**) Cyclic voltammetry (CV) curves of the NTGS from 10~200 mV/s; (**b**) specific capacitances of the NTGS cell as a function of the current density; (**c**) cycling performance of the NTGS cell; (**d**) the Ragone plots of the NTGS cells, compared with those of other recently reported supercapacitors [32,33,34,35,36,37].

It is clear from Figure 3a that the cyclic voltammetry (CV) curves of the NTGS cells (scan rate in the range of 10–200 mV/s) are excellent and exhibit a typical rectangular shape, which confirms the formation of double layer capacitance. The variations in the C*_gra_* values with respect to the current density of the NTGS cell is depicted in Figure 3b. The NTGS cell exhibited stable cycling performance and maintained 88% of its initial capacitance, even after 20,000 cycles at a current density of 5 Ag^−1^ (Figure 3c). Figure 3d shows the Ragone plots of the NTGS cell. It is apparent that the energy density values of the NTGS cells are superior to those of other recently reported supercapacitors.

**Figure 4:**
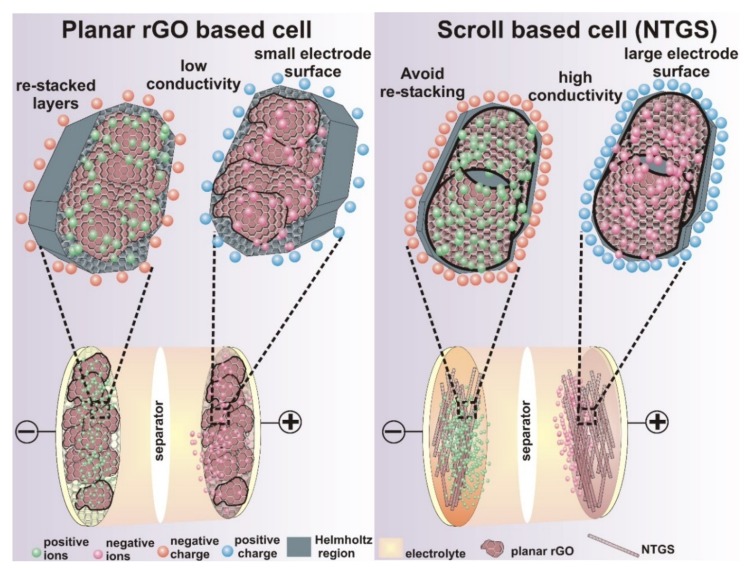
A schematic showing the electrochemical reactions in the rGO cell (**left**) and the NTGS cell (**right**).

The electrochemical impedance spectra (EIS) is a powerful tool for analyzing the internal resistance of the electrode material, and the EIS studies were conducted for the NTGS and pure GO cells separately, at a frequency range between 100 kHz and 100 MHz, with an amplitude of 10 mV in the open circuit condition. The corresponding EIS spectra are presented in Figure S6. The electrode resistance, bulk electrolyte resistance, diffuse layer resistance, and equilibrium differential capacitance can be retrieved directly from Nyquist plots. The Nyquist spectra of both cells consisted of semicircles in the high-to-medium frequency region, and a sloping line in the low-frequency region. The slope of the line represents the diffusion control process in the low-frequency region. The intercept of the semicircle with the real impedance axis (Z′) represents the equivalent series resistance (ESR), which is a combination of electrolyte and contact resistance. The intercept from the vertical line to the *x*-axis gives the total internal resistance of the cell. The ESR values for NTGS and GO cells, calculated [41] from a Nyquist plot, are 9.9 Ω and 19 Ω, respectively (Figure S6). Thus, Figure S6 clarifies that the ESR of the NTGS cell is lower than that of the pure GO cell. The total internal resistance for NTGS and GO cells are 13.5 Ω and 20.8 Ω, respectively (Figure S6). The diameter of the semicircle provides the charge transfer resistance, *R_ct_*, resulting from the diffusion of electrons towards the electrode materials. The lower *R_ct_* of the NTGS cell compared with that of the GO cell indicates the enhancement in the electron transfer process. The scroll structure provides a connected network, and it results in a lower resistance and a shorter electron diffusion pathway, which in turn leads to the enhanced electron transfer process. Furthermore, the open and connected architecture of the conductive scrolls provides a good interface for the electrolyte ions, which increases the electrochemical accessibility of the electrolyte ions into the NTGS electrode. Figure S4 clearly shows that the diffuse layer resistance is lower for NTGS compared to the GO cell. The formation of a narrow Helmholtz region in the NTGS cell is shown as a schematic in Figure 4. The unique structure of the NTGS cells, with their outstanding electrical conductivity, provides a short and rapid transport pathway for electrons, permitting high-capacity and high-energy density at high current densities. The excellent performance of the NTGS cell is suitable for applications that demand high gravimetric energy densities.

## 4. Conclusions

In summary, to enhance the energy density of EDLCs, nitrogen-incorporated, thiol-functionalized, reduced graphene oxide scrolls (NTGS) were synthesized. In the voltage range from 0 to 4.0 V, a supercapacitor fabricated with NTGS powder exhibited high energy densities (206 Wh/kg, 59.74 Wh/L) compared to previously reported values (85–100 Wh/kg), and comparable to those of Li-ion batteries. The superior energy density values of the fabricated NTGS capacitor are attributed to the nitrogen doping, thiol functionalization, and scroll morphology. The NTGS cell also exhibited excellent cycling stability up to 20,000 cycles, and a power density of 32 kW/h (9.8 kW/L). Our strategy in developing NTGS using a combination of nitrogen incorporation, thiol-functionalization, and scroll formation was to offer a significant potential electrode material for energy storage systems, which can also be used to enhance the performance of the other electrode materials, including those for lithium-ion batteries and hybrid supercapacitors.

## Supplementary Materials

The following are available online at http://www.mdpi.com/2079-4991/9/2/148/s1, Figure S1: (**a**–**g**) HRTEM images of an interconnected NTGS powder sample, (**h**) HRTEM image of a single scroll, (**i**) SAED pattern of NTGS sample from the area shown in (**g**). Figure S2: (**a**) Raman spectrum and fitted XPS spectra of (**b**) C*1s*, (**c**) N*1s*, and (**d**) S*2p* GFNS powder sample. The inset of (**a**) shows the Raman spectra of NTGS sample in a different range of vibrational frequencies. Figure S3: The Galvanostatic charge/discharge (GCD) performance of the rGO cell at current densities 1.6 A/g and 1.4 A/g. Figure S4: Ohmic voltage (IR) drop in Galvanostatic charge/discharge curve at a current density of 0.25 A/g. Figure S5: He–1 UPS spectra for the NTGS and rGO samples. Figure S6: Electrochemical impedance spectra of GO and NTGS powder samples. Table S1: The fitted UPS spectra peaks of rGO and NTGS powder samples and their corresponding assignments.
