# Supplementary material for: An Ultra-High-Energy Density Supercapacitor; Fabrication Based on Thiol-functionalized Graphene Oxide Scrolls
Source: Nanomaterials (Basel). 2019 Jan 24;9(2):148. doi: 10.3390/nano9020148 (PMC6409971; doi:10.3390/nano9020148)
Supplement: Supplementary file 1 [file nanomaterials-09-00148-s001.pdf]

Supplementary Information

An Ultra-High-Energy Density Supercapacitor; Fabrication Based on Thiol-functionalized Graphene Oxide Scrolls

Janardhanan. R. Rani [1,†], Ranjith Thangavel [2,†], Se-I Oh [1], Yun Sung Lee [2] and Jae-Hyung Jang [1,3,*]

1 School of Electrical Engineering and Computer Science, Gwangju Institute of Science and Technology, Gwangju 61005, Korea; ranijnair@gmail.com (J.R.R.); ohseia@naver.com (S.-I.O.)
2 Faculty of Applied Chemical Engineering, Chonnam National University, Gwangju 61186, Korea; ranjith.cecri@gmail.com (R.T.); leeys@jnu.ac.kr (Y.S.L.)
3 Research Institute for Solar and Sustainable Energies, Gwangju Institute of Science and Technology Gwangju 61005, Korea
† These authors contributed equally to this work.
* Correspondence: jjang@gist.ac.kr; Tel.:+82-62-715-2209; Fax: +82-62-715-2204

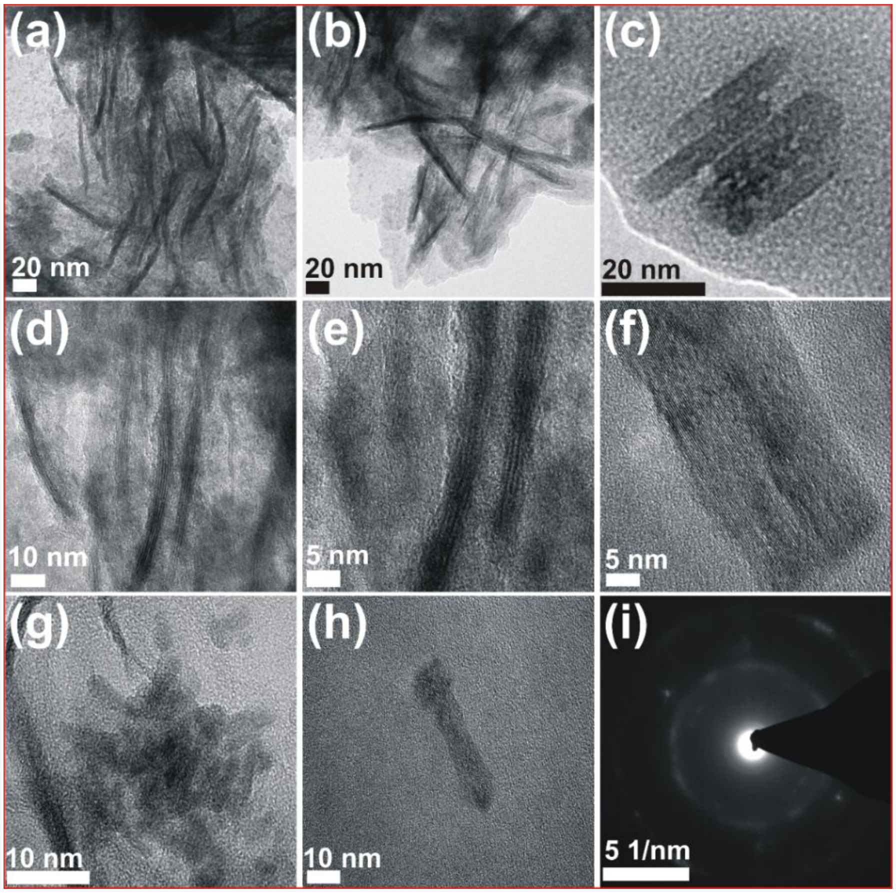

Figure S1. (**a**–**g**) HRTEM images of an interconnected NTGS powder sample, (**h**) HRTEM image of a single scroll, (**i**) SAED pattern of NTGS sample from the area shown in (**g**).

For TEM sample preparation, first, powder samples are dispersed in alcohol followed by sonication to obtain highly dispersed samples. Then a small drop of the dispersed solution was deposited on to the commercially available carbon-coated copper grid. Then leave the grid for 15 min to dry in air. When the solvent is totally evaporated, the grid is fixed on the TEM sample holder for characterization.

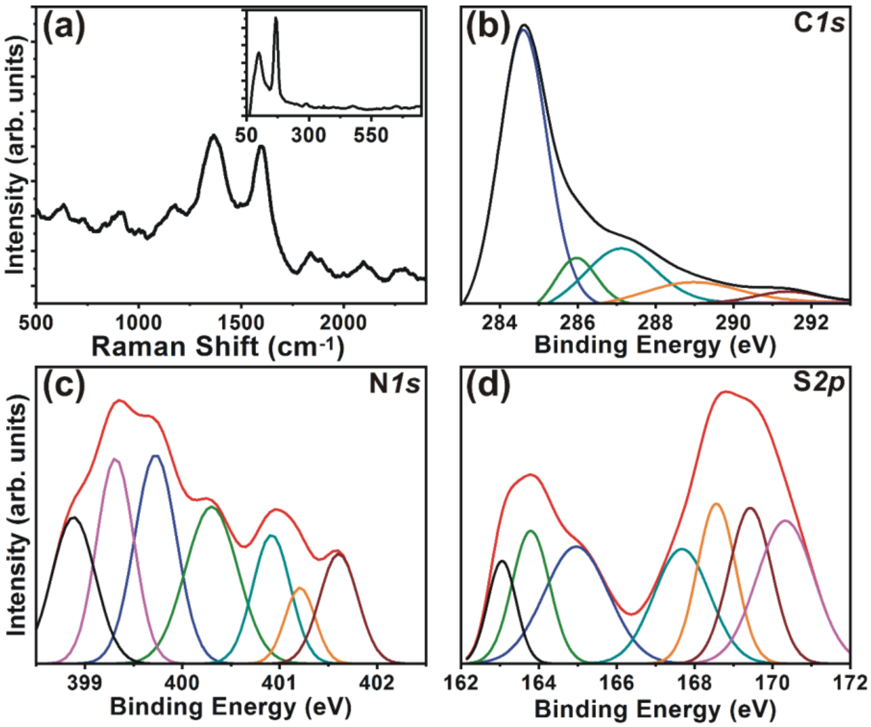

Figure S2. (**a**) Raman spectrum and fitted XPS spectra of (**b**) C*1s*, (**c**) N*1s*, (**d**) S2*p* GFNS powder sample. The inset of (**a**) shows the Raman spectra of NTGS sample in a different range of vibrational frequencies.

The Raman analysis of NTGS powder, exhibiting a G peak at ~1590 cm^{-1}, a D peak at ~1350 cm^{-1} [1,2] as shown in Figure S2a. The Raman spectrum of the NTGS scrolls differs significantly from that of planar graphene. The E_{2g} vibrational modes within aromatic carbon rings result in the formation of the G bands and is induced by a single resonance process, while the D band requires scattering at defect sites in order to conserve momentum [1,2]. Also the broadened D band in NTGS is due to the curvature-induced defect scattering in the scroll structure [2]. Scroll related band is also observed in 920 cm^{-1}, corresponds to the iTA phonons reported for graphite whiskers and CNTs [1,2]. The band at 1180 cm^{-1} is due to N–C stretching mode [1,2]. The peak at 1800 cm^{-1} is a combination of iTA and LO phonons and a peak around 2000 cm^{-1} is a combination of the oTO (out-of-plane tangential optical) and LO phonon mode around the K point in the Brillouin zone [1,2]. These modes are known as M band. oTO phonon of graphene at the Γ point in the two-dimensional Brillouin zone is not Raman active mode and is active in the present study due to the curvature induced scroll structure. Band observed at ~2280 cm^{-1} is a combination mode (combination of the iTO and iTA phonons) around the K point [1,2]. Thus Raman spectrum of NTGS powder clearly confirms the strong bonding between Carbon, Sulphur, and Nitrogen and side wall functionalization of the scroll structure.

A typical C 1s XPS spectrum (Figure S2b) can be de convoluted into several peaks including C–C (~284.5 eV), C–N (~285.6 eV), CO/CS (~286.6 eV), O–C–O (~289.2 eV), and π-π* (~290.4 eV) [3]. The C1s XPs spectrum clearly confirms the bonding between C, N and Sulphur. The high resolution of N1s scanning spectrum (FigureS2c) was deconvoluted into seven individual components such as 398.8 eV (pyridinic N), 399.2 eV, 399.7 eV (pyrrolic N), 400.3 eV, 400.9 eV (graphitic N), 401.2 eV pyridinic (N^+–O^-) and 401.6 eV (oxidized nitrogen) [4]. The binding energies at 399. 2 eV and 400.3 eV may be due to N–C–S bonding [4]. Thus N1s spectrum clearly shows C–N bonding, which can produce lone pairs in the sample. S2p XPS spectrum (Figure S3d) can be de convoluted into several peaks including spin orbit doublets of S 2p3/2 and S 2p1/2. The two prominent peaks at 163.7 eV and 165.02 correspond to S 2p3/2 and S 2p1/2 of the C–S–C covalent bond of thiophene–S caused by a spin-orbit coupling respectively [5]. Other peaks observed, corresponds to 163.03 (sulphide), 167.6 eV (thiosulphate), 168.5 eV (S–O), 169.4 eV

(sulphate), 170.03 eV (SOx groups) [5]. S2p XPS spectra clearly confirms the strong chemical interaction of Sulphur with the carbon material.

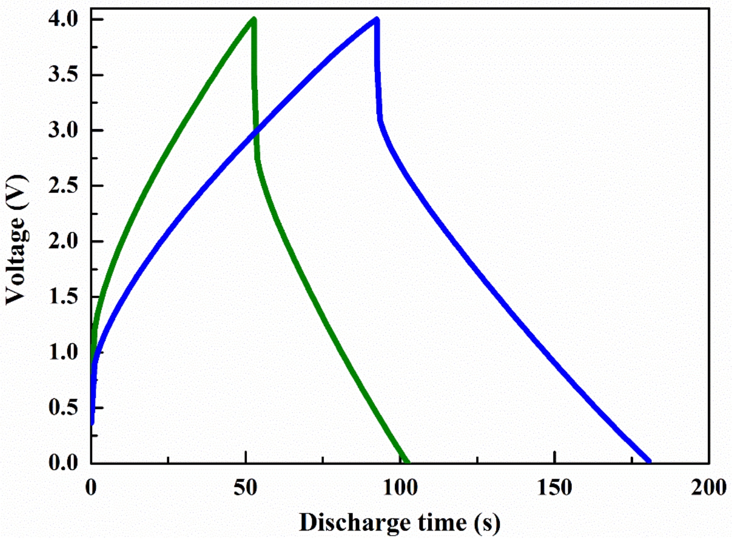

Figure S3. The GCD performance of the rGO cell at current densities 1.6 A/g and 1.4 A/g.

The GCD performance of the rGO cell is shown in the Figure S3.

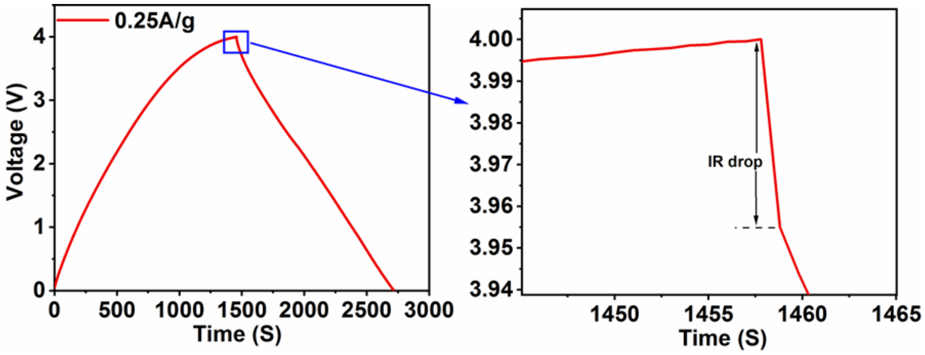

Figure S4. IR drop in Galvanostatic charge-discharge curve at a current density of 0.25 A/g.

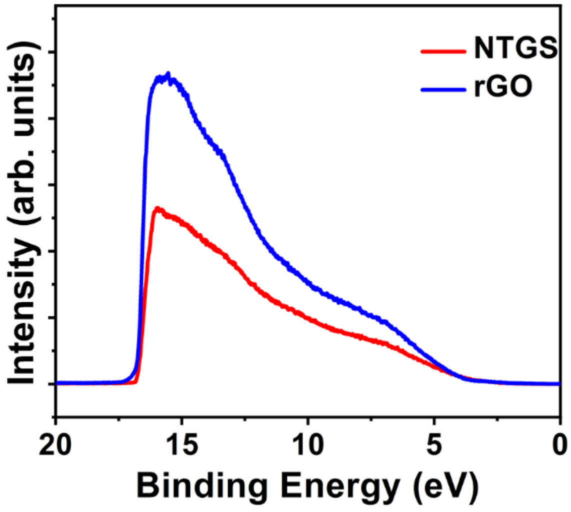

Figure S5. He-1 UPS spectra for the NTGS and rGO samples.

The fitted UPS spectra peaks and their corresponding assignments are shown in Table S1.

Table S1. The fitted UPS spectra peaks of rGO and NTGS powder samples and their corresponding assignments.

sample	2p π–σ (eV)	2p σ (eV)	C2s-C2p (eV)	C2p-3p eV)	C2p-S3s (eV)	C2p-2p (eV)	2s (eV)	Ref.
rGO	5.4	7.3	9.87	-			14.3	[6]
NTGS	5.46	6.97	8.6 (N lone pair also)	10.5	11.8	13.7	14.64	[6]

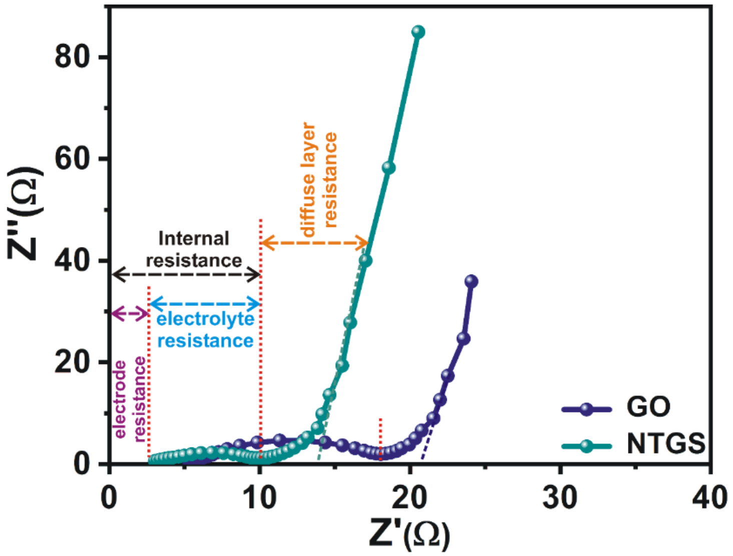

Figure S6. Electrochemical impedance spectra of GO and NTGS powder samples.

References

1. Ferrari, A.C.; Robertson, J. Interpretation of Raman spectra of disordered and amorphous carbon. *Phys. Rev. B* **2000**, *61*, 14095.
2. Rani, R.J.; Oh, S.-I.; Jang, J.-H. Raman Spectra of Luminescent Graphene Oxide (GO)-Phosphor Hybrid Nanoscrolls. *Materials* **2015**, *8*, 12.
3. Rani, J.R.; Lim, J.; Oh, J.; Kim, J.-W.; Shin, H.S.; Kim, J.H.; Lee, S.; Jun, S.C. Epoxy to Carbonyl Group Conversion in Graphene Oxide Thin Films: Effect on Structural and Luminescent Characteristics. *J. Phys. Chem. C* **2012**, *116*, 19010–19017.
4. Liu, X.; Zhou, W.; Yang, L.; Li, L.; Zhang, Z.; Ke, Y.; Chen, S. Nitrogen and sulfur co-doped porous carbon derived from human hair as highly efficient metal-free electrocatalysts for hydrogen evolution reactions. *J. Mater. Chem. A* **2015**, *3*, 8840–8846.
5. Li, R.; Li, J.; Qi, K.; Ge, X.; Zhang, Q.; Zhang, B. One-step synthesis of 3D sulfur/nitrogen dual-doped graphene supported nano silicon as anode for Li-ion batteries. *Appl. Surf. Sci.* **2018**, *433*, 367–373.
7. Umebayashi, T.; Yamaki, T.; Yamamoto, S.; Miyashita, A.; Tanaka, S.; Sumita, T.; Asai, K. Sulfur-doping of rutile-titanium dioxide by ion implantation: Photocurrent spectroscopy and first-principles band calculation studies. *J. Appl. Phys.* **2003**, *93*, 5156.
